# Blood Thixotropy in Patients with Sickle Cell Anaemia: Role of Haematocrit and Red Blood Cell Rheological Properties

**DOI:** 10.1371/journal.pone.0114412

**Published:** 2014-12-11

**Authors:** Jens Vent-Schmidt, Xavier Waltz, Marc Romana, Marie-Dominique Hardy-Dessources, Nathalie Lemonne, Marie Billaud, Maryse Etienne-Julan, Philippe Connes

**Affiliations:** 1 Inserm UMR 1134, Hôpital Ricou, CHU de Pointe-à-Pitre, 97157, Pointe-à-Pitre, Guadeloupe, France; 2 Laboratory of Excellence GR-Ex « The red cell: from genesis to death », PRES Sorbonne Paris Cité, 75015, Paris, France; 3 Laboratory ACTES (EA 3596), Department of Physiology, French West Indies and Guiana University, Pointe-à-Pitre, Guadeloupe, France; 4 Unité Transversale de la Drépanocytose, CHU de Pointe-à-Pitre, 97157, Pointe-à-Pitre, Guadeloupe, France; 5 Institut Universitaire de France, Paris, France; 6 Laboratoire CRIS EA647, Université Claude Bernard Lyon 1, 69100, Villeurbanne, France; Emory University/Georgia Insititute of Technology, United States of America

## Abstract

We compared the blood thixotropic/shear-thinning properties and the red blood cells’ (RBC) rheological properties between a group of patients with sickle cell anaemia (SS) and healthy individuals (AA). Blood thixotropy was determined by measuring blood viscosity with a capillary viscometer using a “loop” protocol: the shear rate started at 1 s^−1^ and increased progressively to 922 s^−1^ and then re-decreased to the initial shear rate. Measurements were performed at native haematocrit for the two groups and at 25% and 40% haematocrit for the AA and SS individuals, respectively. RBC deformability was determined by ektacytometry and RBC aggregation properties by laser backscatter versus time. AA at native haematocrit had higher blood thixotropic index than SS at native haematocrit and AA at 25% haematocrit. At 40% haematocrit, SS had higher blood thixotropic index than AA. While RBC deformability and aggregation were lower in SS than in AA, the strength of RBC aggregates was higher in the former population. Our results showed that 1) anaemia is the main modulator of blood thixtropy and 2) the low RBC deformability and high RBC aggregates strength cause higher blood thixotropy in SS patients than in AA individuals at 40% haematocrit, which could impact blood flow in certain vascular compartments.

## Introduction

Blood is a non-newtonian fluid with visco-elastic, shear thinning and thixotropic properties. A thixotropic fluid is a fluid whose viscosity is a function not only of the shearing stress, but also of the previous history of motion within the fluid [1]. The viscosity usually decreases with the length of time the fluid has been in motion. Increased blood thixotropy has been reported in patients with coronary artery disease [2]. More recently, Franco et al. [3] reported increased blood thixotropy in patients with Gaucher Disease. In both cases the increased blood thixotropy resulted from enhanced RBC aggregation [2], [3], and was suspected to participate in the cardiovascular and microcirculatory disorders observed in these two diseases.

Patients with sickle cell anaemia (SS) have severe haematological and haemorheological abnormalities [4]. Several studies demonstrated the key role of abnormal haemorheology in several acute and chronic complications in SS [4], [5], [6], [7], [8], [9], [10], [11], [12], [13], [14], [15], [16], [17], [18], [19], [20], [21], [22]. However, although an important number of studies reported blood viscosity data in SS, there is no published data on the thixotropic properties of blood in this population. The present study provides information about the blood thixotropic and shear-thinning properties determined at different haematocrits and red blood cells’ (RBC) rheological properties of SS patients compared to healthy individuals (AA).

## Materials and Methods

### Patients

Blood from 18 SS patients and 8 AA were sampled in EDTA tubes. AA subjects were healthy individuals: i.e., without hemoglobin disorders, anemia or cardiac, lungs or metabolic disease. The SS patients recruited are regularly followed up by the Sickle Cell Unit of the Academic Hospital of Pointe-à-Pitre (Pointe-à-Pitre, Guadeloupe). Hemoglobin analysis and quantifications were performed using isoelectrofocusing (Multiphor II System, GE HEALTH CARE, Buck, UK), citrate agar electrophoresis and cation-exchange high performance liquid chromatography (VARIANT, Bio-Rad Laboratories, Hercules, CA, USA). All participants were aged ≥18 yrs old. Sickle cell patients were in clinical steady state at the time of the study (*i.e.,* without vaso-occlusive crisis, acute medical complication within the last month or blood transfusion/phlebotomies within the last 3 months). Participants provide informed written consent to participate. The study was conducted in accordance to the Declaration of Helsinki, and approved by the Regional Ethics Committee (CPP Sud/Ouest Outre Mer III, Bordeaux, France, registration number: 2010-A00244-35).

### Determination of plasma viscosity and blood thixotropy

All haemorheological measurements were carried out by following the recent guidelines for international standardization in blood rheology techniques/measurements and interpretation [23].

Plasma is a Newtonian fluid: its viscosity was measured at 106 s^−1^ and 37°C using a capillary viscometer (Vilastic bioprofiler, Vilastic Scientific, Austin, TX). Blood viscosity was determined at native haematocrit (Hct) and adjusted Hct (25% for AA and 40% for SS) using autologous plasma, at 37*°*C and at various shear rates using the same capillary viscometer oscillating at a frequency of 2 Hz [24]. Hct was measured by microcentrifugation as recommended [23]. We used a “loop” protocol where the shear rate started at 1 s^−1^ and increased progressively (every 5 seconds) to 922 s^−1^ and then re-decreased progressively to the initial shear rate (see [1], [24] for details). This hysteresis loop protocol allows the characterization of the blood thixotropic properties. The difference between the two blood viscosity curves for a given shear rate was calculated and plotted vs shear rate: the area under the curve was calculated and corresponded to the thixotropic index [1].

### Ektacytometry

RBC deformability was determined at 37°C at nine shear stresses ranging from 0.30 to 30 Pa by laser diffraction analysis (ecktacytometry), using the Laser-assisted Optical Rotational Cell Analyzer (LORCA, RR Mechatronics, Hoorn, The Netherlands). The system has been described in detail elsewhere [23]. Briefly, 25 µl of prepared blood suspension was mixed with 5 ml polyvinylpyrrolidone (PVP; viscosity = 30 cP, RR Mechatronics, Hoorn, The Netherlands) and sheared into the glass Couette system. The diffraction pattern was analyzed by the computer and an elongation index was calculated. An increase of the elongation index indicates greater RBC deformability. The value of 3 Pa is often considered to be a threshold between low/moderate shear and high shear stress values. Under 3 Pa, RBC deformability is more dependent on the ability of RBC membrane to deform under shear stress whereas above 3 Pa, RBC deformability mainly depends on the internal viscosity of the cells [23].

### Determination of RBC aggregation properties

RBC aggregation properties were determined at 37°C by laser backscatter versus time, using the Laser-assisted Optical Rotational Cell Analyzer (LORCA, RR Mechatronics, Hoorn, The Netherlands), after adjustment of the Hct to 40% with autologous plasma [23], [25]. Blood was inserted into the Couette system of the LORCA and subjected to high shear for 2 s (800 s^−1^) to dissociate pre-existing RBC aggregates. Then, shearing was stopped abruptly and the changes in laser backscatter intensity were monitored for 2 minutes (syllectogram) by a photodiode sensor incorporated into the LORCA. The syllectogram patterns exhibit three exponential phases with the immediate short rising phase corresponding to the time needed for RBCs to recover their resting shape. The amplitude and the half-time of the whole syllectogram were used to calculate the RBC aggregation index (AI). The RBC disaggregation threshold (i.e., RBC aggregates strength) was determined using a re-iteration procedure [25]: 7 separate pre-defined shear rates between 7.5 s^−1^ and 800 s^−1^ were applied with or without alternating disaggregation shear rate to the RBC suspension, to locate the minimal shear rate needed to prevent RBC aggregation.

### Statistical analyses

The different rheological and hemorheological terms used in this article are defined in the Table 1. Unpaired student t test or a one-way ANOVA with Newman-Keuls test for post hoc comparisons were used to compare haemorheological parameters between the two groups or the different haematocrit conditions, respectively. Statistical significance was determined by p value <0.05. Analyses were conducted using Statistica (Version 8.0; StatSoft, Tulsa, OK, USA) and data were reported as mean ± SD.

**Table 1:** Definition of the rheological and hemorheological terms.

Terms	Definition
Newtonian fluid	A Newtonian fluid is a fluid whose viscosity remains constant whatever the level of the applied force or the time during which the force is applied
Non-newtonian fluid	A non-newtonian fluid is a fluid whose viscosity changes with the level and/or the time of the force application
Shear rate	This is the velocity gradient measured across the diameter of a fluid-flow channel. In vascular physiology, shear rate depends on blood flow and the vessel radius
Shear stress	This is the external force acting on an object or surface parallel to the slope or plane in which it lies; the stress tending to produce shear. In hemorheology, shear stress is the product of blood viscosity by shear rate
Shear thinning	This is the rheological behavior of a fluid where viscosity decreases when the force applied increases
Thixotropy	A thixotropic fluid is a fluid whose viscosity is a function not only of the shearing stress, but also of the previous history of motion within the fluid
Blood viscosity	It is a measure of the resistance of blood (i.e., plasma + blood cells) to flow
Plasma viscosity	It is a measure of the resistance of plasma to flow
Red blood cell deformability	This is the ability of red blood cell to change its shape under a given level of applied force, without rupture. It depends on the elasticity and integrity of the membrane, the cytosolic viscosity and the ratio surface/volume of the cell
Red blood cell elasticity	Red blood cell (membrane) elasticity is the ability of red blood cell to recover its initial shape after a deformation obtained for a given force. It depends on the membrane integrity
Red blood cell aggregation	Red blood cells are able to form reversible aggregates. These aggregates depend on the ability of the cells to aggregate (i.e., aggregability) and on plasma factors that may favor the formation of these aggregates (such as fibrinogen)
Red blood cell disaggregation threshold	This is the force needed to disperse RBC aggregates

## Results

### RBC deformability and aggregation properties

**Figure 1:**
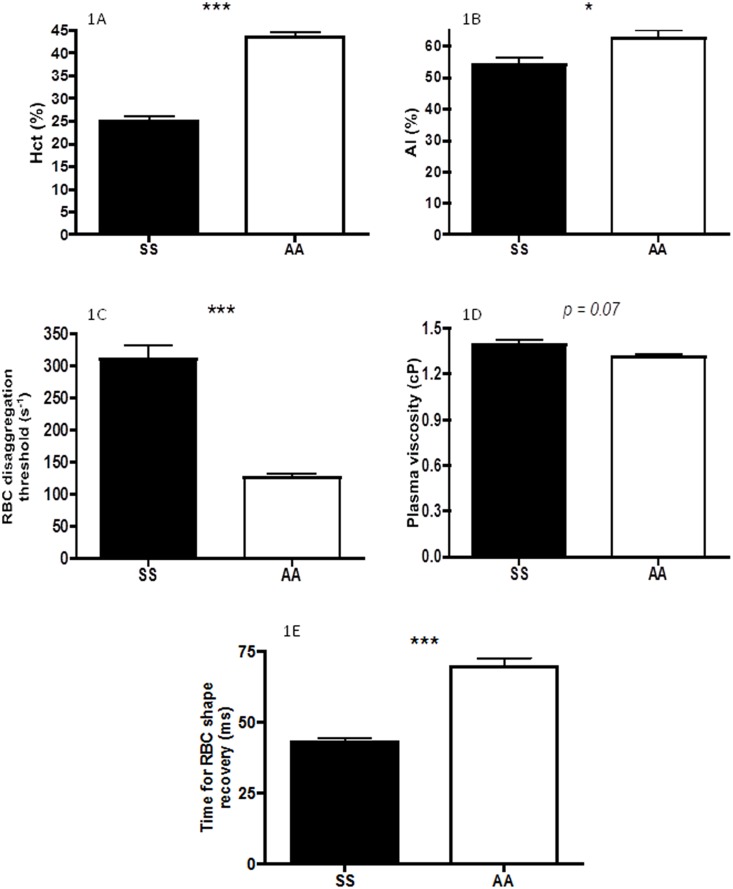
A–E: Haematocrit (Hct; 1A), red blood cell aggregation index (AI; 1B), red blood cell aggregates strength (disaggregation threshold; 1C), plasma viscosity (1D) and time for red blood cell shape recovery (1E) in patients with sickle cell anaemia (SS) and healthy individuals (AA). Significant difference: *p<0.05; ***p<0.001.

As expected, native Hct was lower in SS than in AA (Fig. 1A, p<0.001). While RBC aggregation (AI) was lower in SS than in AA (p<0.05), the RBC disaggregation threshold was 3 fold higher in the former group (p<0.001). Plasma viscosity tended to be slightly greater in the SS group (p = 0.07). At all shear stress levels, except the lowest one, SS had lower RBC deformability than AA (Table 2, p<0.001). The time for RBC shape recovery calculated from the syllectogram was shorter in SS than in AA (Fig. 1E, p<0.001).

**Table 2:** Red blood cell (RBC) deformability at different shear stresses in patients with sickle cell anaemia (SS) and healthy individuals (AA).

	AA	SS
RBC deformability at 0.3 Pa	0.02±0.02	0.02±0.03
RBC deformability at 0.53 Pa	0.05±0.02	0.03±0.02***
RBC deformability at 0.95 Pa	0.12±0.01	0.05±0.03***
RBC deformability at 1.69 Pa	0.22±0.02	0.12±0.04***
RBC deformability at 3.0 Pa	0.32±0.02	0.18±0.04***
RBC deformability at 5.33 Pa	0.42±0.02	0.25±0.05***
RBC deformability at 9.49 Pa	0.49±0.02	0.31±0.07***
RBC deformability at 16.87 Pa	0.54±0.02	0.36±0.07***
RBC deformability at 30 Pa	0.59±0.01	0.41±0.09***

Significant difference: ***p<0.001.

### Blood thixotropy

**Figure 2:**
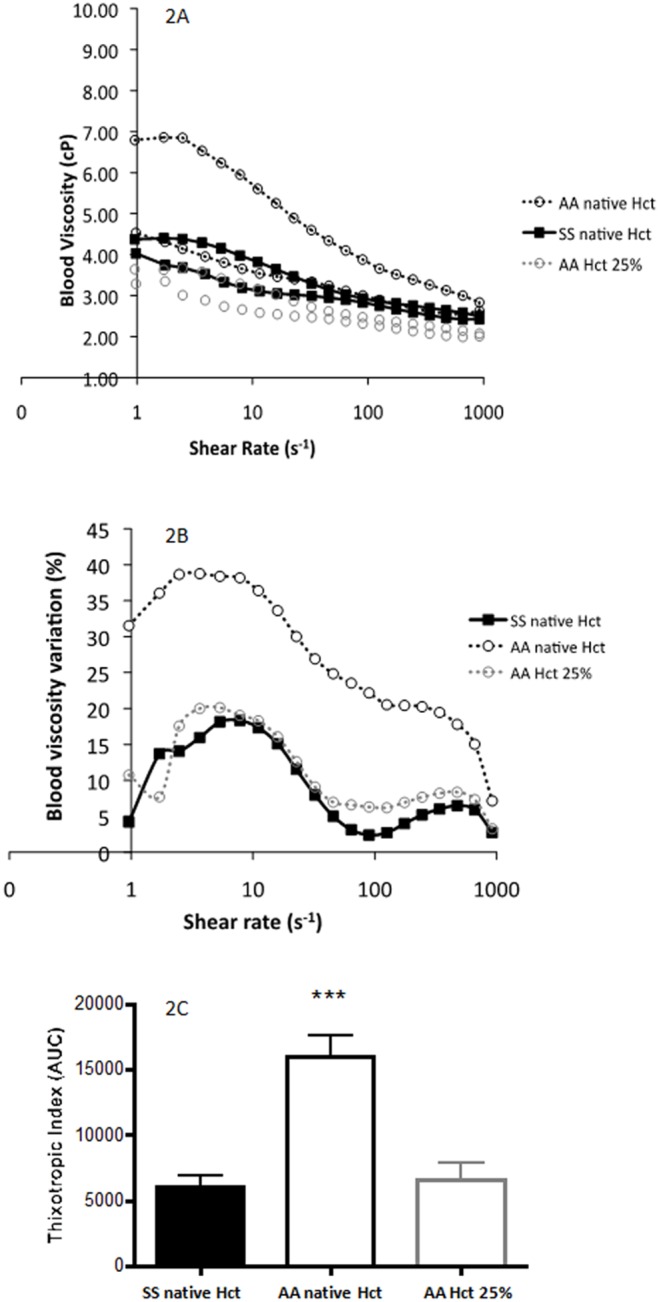
A–C: Blood viscosity hysteresis loop (2A), differences between the two blood viscosity curves of the loop obtained on Fig. 3A (2B) and blood thixotropic index in patients with sickle cell anaemia (SS) at native haematocrit and healthy individuals (AA) at both native and 25% haematocrit. Significantly from AA at native haematocrit: ***p<0.001.

Fig. 2A represents the mean hysteresis loop for SS at native Hct and for AA at both native and 25% Hct. On the whole, blood viscosity in SS at native Hct was higher than blood viscosity of AA at 25% Hct (p<0.01). In contrast, blood viscosity in AA at native Hct was higher than blood viscosity of SS at native Hct and AA at 25% Hct (p<0.001). Fig. 2B shows the results of the difference between the two blood viscosity curves obtained during the loop protocol for each group: the area under the curve was calculated and corresponded to the thixotropic index (Fig. 2C). Our results demonstrated that AA at native Hct had higher thixotropic index than both AA at 25% Hct and SS at native Hct (p<0.001) while thixotropic index was similar for these two last conditions.

**Figure 3:**
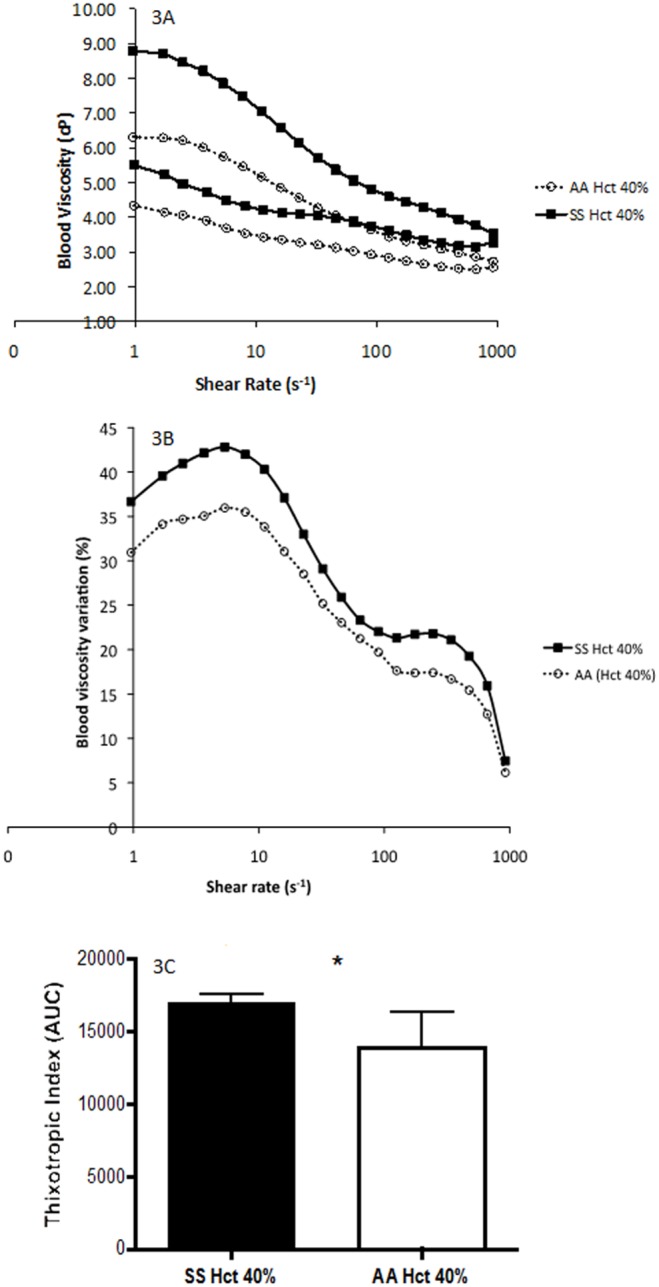
A–C: Blood viscosity hysteresis loop (3A), differences between the two blood viscosity curves of the loop obtained on Fig. 3A (3B) and blood thixotropic index in patients with sickle cell anaemia (SS) and healthy individuals (AA) at 40% haematocrit. Significant difference: *p<0.05.

Figs. 3A and 3B depict the mean hysteresis loop and the difference in blood viscosity between the two curves for SS and AA at 40% Hct, respectively. Blood viscosity at 40% Hct was higher in SS than in AA (p<0.001). We observed higher thixotropic index in SS than in AA (Fig. 3C, p<0.05).

## Discussion

For the first time, our results demonstrated that low Hct (anaemia) was responsible for the loss of blood thixotropy and shear thinning property in SS. In contrast, at adjusted Hct (40%), SS exhibited higher blood thixotropic index than AA.

The phenomenon of thixotropy in a liquid results from the microstructure of the liquid system. Thixotropy may be explained as a consequence of aggregation of suspended particles. If the suspension is at rest, the particles can aggregate. On the other hand, if the suspension is sheared, the weak physical bonds among particles are disrupted, and the network breaks down into separate aggregates which can further disintegrate into smaller fragments [1]. This phenomenon can be observed in blood where RBCs form aggregates at low shear rate [24], [26]. RBC aggregation in blood is a reversible process with progressive disaggregation occurring with either increasing shearing time or shear rate level [24], [26].

As mentioned above, increased blood thixotropy has been reported in patients with coronary artery disease [2] and Gaucher Disease [3]. It was proposed that the increased blood thixotropy in both cases [2], [3] could participate to the cardiovascular and microcirculatory disorders observed in these diseases. Our study is the first to investigate blood thixotropy in sickle cell patients. We showed that blood thixotropy at native Hct was lower in SS compared to AA. The Hct of AA was 2 fold greater than in SS, thus allowing more frequent contact between adjacent RBCs to form aggregates. Indeed, chronic anaemia is the main cause of the low thixotropic index found in SS. Besides, when whole blood of AA was diluted with autologous plasma to lower Hct to the same level found in SS (i.e., 25%), the thixotropic index decreased and became similar to that of SS.

In contrast, when Hct was adjusted to 40% in both groups, blood thixotropic index was higher in SS than in AA. The fact that the blood thixotropic index was higher in SS than in AA at 40% Hct but not at 25% Hct indicates that anaemia compensates, to some extent, for the other RBC rheological abnormalities. The formation of RBC aggregates is more difficult at 25% Hct than at 40%. Indeed, the impact of the RBC aggregation abnormalities on blood thixotropy in SS is of less importance at native Hct than at 40% Hct.

The greater blood thixotropy in SS at 40% Hct was not caused by higher RBC aggregation since the aggregation index was lower in this group compared to AA. The low RBC aggregation in SS is mainly caused by the presence of very rigid poorly aggregable RBCs [4], [17], [27]. However, although RBC aggregation was lower in SS, the strength of RBC aggregates (i.e. RBC disaggregation threshold) was greater than that of AA; a finding previously reported [22], [28], and more particularly in SS with glomerulopathy [18]. This finding indicates that with progressively increasing shear rate, the less frequent and/or less large RBC aggregates in SS persist longer than in AA, thereby impacting blood viscosity at low but also at moderate and high shear rates. At high shear rate, blood viscosity is mainly dependent on the ability of RBCs to deform [26]. The lower RBC deformability in SS resulted in higher blood viscosity at high shear rate, in comparison with AA.

During the descending phase of a loop protocol, blood viscosity first depends on the ability of RBCs to recover their initial shape, and then, at low shear rate, blood viscosity depends on the ability of RBCs to re-aggregate [24]. The time for RBC shape recovery was shorter in SS compared to AA but that does not indicate better RBC membrane elasticity. Instead, it is the consequence of the poor deformation of RBCs under high shear rate making them to quickly return to their resting rigid shape when shear rate decreases or stops. Indeed, the quick return of SS RBCs to their rigid shape affected blood viscosity of patients to a greater extent than in AA in the high shear zones. As shear rate progressively decreases to lower values, RBC aggregates re-form but this process was of lower magnitude in SS than in AA and may explain why, for example, the difference in blood viscosity during the loop protocol at 6 s^−1^ peaked at 43% in SS while it peaked at 36% in AA.

In summary, the lower blood thixotropy found at native Hct in SS could be a protective characteristic against deleterious effects on the vascular function. Nevertheless, Hct varies with the diameter of vessels, a phenomenon called Fahraeus effect [29], [30]. Indeed, it may be possible that, in vivo and in certain vascular compartments, blood thixotropy could be higher in SS than in AA, and impact the blood flow structure and vascular resistance. Unfortunately, the size of the SS group was too small to investigate the associations between blood thixotropy and clinical severity/complications. Further studies are clearly needed on large cohorts to test the clinical importance and relevance of blood thixotropic characteristics in the pathology of sickle cell anaemia.
